# Association between high cardiac output at altitude and acute mountain sickness: preliminary study on Mt. Fuji

**DOI:** 10.1186/s40101-023-00322-7

**Published:** 2023-04-13

**Authors:** Takeshi Ebihara, Kentaro Shimizu, Yumi Mitsuyama, Hiroshi Ogura, Jun Oda

**Affiliations:** Department of Traumatology and Acute Critical Medicine, Osaka University Graduate School of Medicine, 2-15 Yamadaoka, Suita City, Osaka, 565-0871 Japan

**Keywords:** Cardiac function, Mt. Fuji, Mt. Fuji research station, Non-invasive hemodynamic monitor

## Abstract

**Background:**

Acute mountain sickness (AMS) affects around 30% of people climbing Mt. Fuji, but its pathogenesis is incompletely understood. The influence of a rapid ascent to high altitude by climbing and summiting Mt. Fuji on cardiac function in the general population is unknown, and its association with altitude sickness has not been clarified.

**Methods:**

Subjects climbing Mt. Fuji were included. Heart rate, oxygen saturation, systolic blood pressure, cardiac index (CI) and stroke volume index were measured multiple times at 120 m as baseline values and at Mt. Fuji Research Station (MFRS) at 3,775 m. Each value and its difference from the baseline value (Δ) of subjects with AMS (defined as Lake Louise Score [LLS] ≥ 3 with headache after sleeping at 3,775 m) were compared with those of non-AMS subjects.

**Results:**

Eleven volunteers who climbed from 2,380 m to MFRS within 8 h and stayed overnight at MFRS were included. Four suffered AMS. Compared with the non-AMS subjects, CI in the AMS subjects was significantly higher than that before sleeping (median [interquartile range]: 4.9 [4.5, 5.0] vs. 3.8 [3.4, 3.9] mL/min/m^2^; *p* = 0.04), and their ΔCI was significantly higher before sleeping (1.6 [1.4, 2.1] vs. 0.2 [0.0, 0.7] mL/min/m^2^; *p* < 0.01) and after sleeping (0.7 [0.3, 1.7] vs. -0.2 [-0.5, 0.0] mL/min/m^2^; *p* < 0.01). ΔCI in the AMS subjects dropped significantly after sleeping versus before sleeping (3.8 [3.6, 4.5] vs. 4.9 [4.5, 5.0] mL/min/m^2^; *p* = 0.04).

**Conclusions:**

Higher values of CI and ΔCI were observed at high altitude in the AMS subjects. A high cardiac output might be associated with the development of AMS.

## Background

Mt. Fuji, the highest mountain in Japan, was registered as a UNESCO World Heritage site in 2013. Hundreds of thousands of travelers climb the mountain every year. Travelers usually arrive at an altitude of around 2,300 m by bus or other transportation and climb to the summit at 3,776 m within a short time. This rapid elevation causes about 30% of the climbers to suffer from acute mountain sickness (AMS) [1, 2].

AMS was reported as a nonspecific condition in which headache, nausea, fatigue, respiratory distress and sleep disturbance can occur after a short ascent to an altitude of 2,500 m or more [3]. AMS is defined at least as headache and total Lake Louise Score (LLS) of > 3. Headache, nausea, fatigue, respiratory disease and sleep disturbance are not parallel [4]. Moreover, Roach et al. [5] recently revisited the LLS and excluded sleep disturbance as a factor. AMS symptoms are exacerbated during the first night at high altitude and improve after one to two days or by descending to a lower altitude [6, 7]. Most cases are not fatal, but cerebral edema and pulmonary edema may occur in some and become critical [8].

Previous studies have investigated numerous factors as potential risks for AMS. Altitude attained [9] and rate of ascent [7] were established as risk factors. A study of the prevalence of AMS on Mt Fuji showed no association with sex or age [1], and a meta-analysis showed no association between age and risk of AMS [10]. A higher body mass index was reported as a possible risk factor [11]. The relation of various vital signs with AMS was also assessed. Although the association between heart rate (HR) variability and AMS remains controversial [12, 13], a low SpO_2_ and higher respiratory rate at high altitudes are reportedly associated with developing AMS [14, 15]. Sleeping at high altitudes may also exacerbate symptoms of AMS [2, 16, 17].

The increase in cardiac output observed when moving to high altitude is considered an adaptation to maintain O_2_ delivery [18, 19]. However, changes in cardiac function caused by rapid ascent to high altitude by climbing and summiting Mt. Fuji and their relationship with altitude sickness are not clarified. We hypothesized that in subjects not showing increased cardiac output at high altitudes, AMS might be a result of tissue hypoxia. We thus aimed to evaluate cardiac function using a non-invasive hemodynamic monitoring device and to clarify the relationship between cardiac function and AMS.

## Methods

### Subjects

**Fig. 1:**
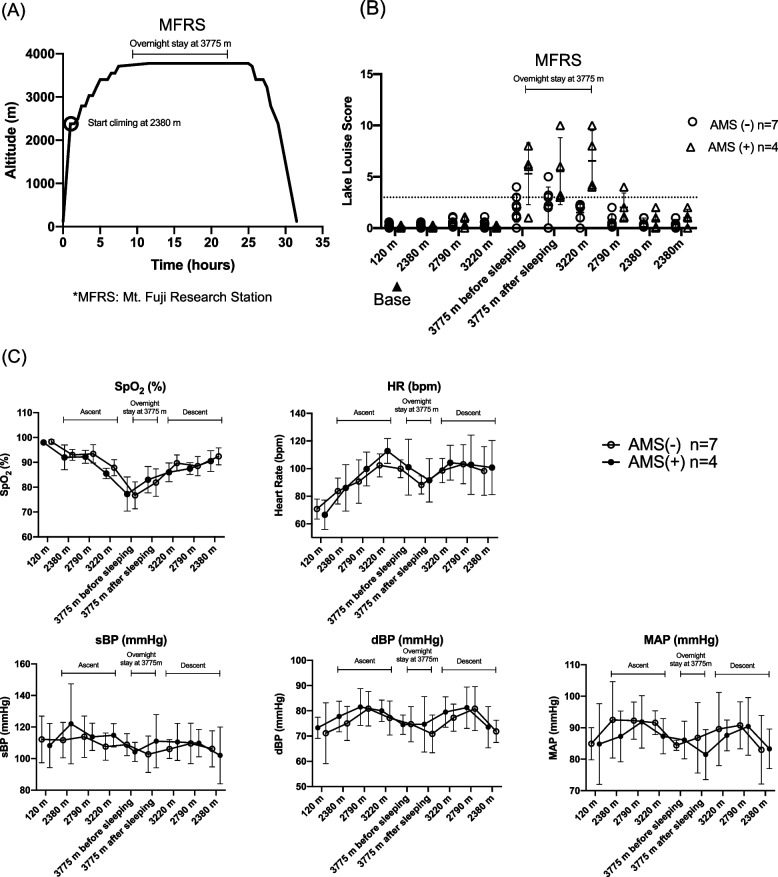

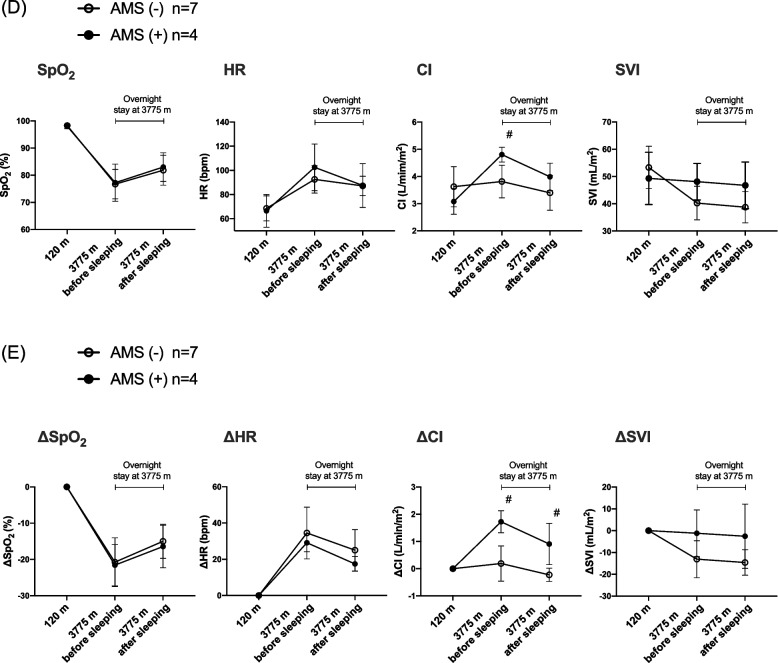
Time course of study and changes of LLS and physiological parameters. **A** The X axis shows the times after departing from Fujinomiya city at 120 m. The Y axis shows the altitude. **B** In this study, AMS was defined as a LLS ≥ 3 with headache after sleeping at 3775 m. Dotted line indicates LLS of 3. **C** Change in SpO_2_, HR, sBP, dBP and MAP. All values are expressed as the mean ± SD. **D** SpO_2_, HR, CI and SVI values of the AMS and non-AMS subjects at 120 m and 3775 m. #Indicates a statistically significant difference between the AMS and non-AMS subjects (*p* < 0.05). **E** The ΔSpO_2_, ΔHR, ΔCI and ΔSVI values of the AMS and non-AMS subjects at 120 m and 3775 m. #Indicates a statistically significant difference between the AMS and non-AMS subjects (*p* < 0.05). All values are expressed as the mean ± SD. *AMS* acute mountain sickness, *CI* cardiac index, *dBP* Diastolic blood pressure, *HR* heart rate, *LLS* Lake Louise Score, *MAP* Mean arterial pressure*, MFRS* Mt. Fuji Research Station, *sBP* Systolic blood pressure, *SD* standard deviation, *SVI* stroke volume index

This study included 11 healthy subjects who climbed mountains of > 2000 m once or twice per year. None had a medical history including respiratory and cardiovascular disease, took any medications, or smoked. All had a body mass index < 25 kg/m^2^. They had travelled from Fujinomiya city (120 m) to the starting point (2,380 m) of the climb by car in early morning. They climbed to Mt. Fuji Research Station (MFRS) at 3,775 m, very near the 3,776-m summit of Mt. Fuji, within 8 h. After staying overnight at the MFRS, they descended to the starting point (Fig. 1A). The AMS subjects in this study were defined as those with an LLS [5] ≥ 3 with headache after sleeping at the MFRS.

This study was performed according to the principles of the Declaration of Helsinki and received approval from the institutional review board of Osaka University Hospital (Permit Number: 18090). Informed consent was obtained from all subjects.

### Measurements

The symptoms of AMS were scored based on the LLS, with its maximum point sum of 12: headache (0 = without headache, 1 = mild headache, 2 = moderate headache, 3 = severe headache); dizziness (0 = without dizziness, 1 = mild dizziness, 2 = moderate dizziness, 3 = severe dizziness); gastrointestinal symptoms (0 = without and 1 = with gastrointestinal symptoms); and fatigue (0 = without and 1 = with fatigue) [17].

SpO_2_, HR and blood pressure were measured by a pocket pulse oximeter (LUKLA-2800 ma; UBIX Corporation, Philippines) and portable blood pressure monitor (HEM-7130; Omron Healthcare Co. Ltd, Japan), respectively. Mean arterial blood pressure (MAP) was calculated by (2 × Diastolic blood pressure + Systolic blood pressure) / 3. These parameters were evaluated at 120 m as baseline values, three times during climbing, two times (before and after sleeping) during the stay at the MFRS and three times during the descent. Cardiac index (CI) and stroke volume index (SVI) were measured by a non-invasive hemodynamic monitoring device (ClearSight system; Edwards Lifesciences, Irvine, CA) at 120 m as baseline values and twice (before and after sleeping) during the stay at the MFRS. This device began to be used in the intensive care unit in 2015. It measures the arterial pressure curve via a sensor wrapped around the finger and calculates a beat-to-beat stroke volume (SV) by dividing the area under the systolic portion of the arterial pressure curve by aortic input impedance [20]. Beat-to-beat cardiac output is calculated by multiplying beat-to-beat SV by instantaneous HR. Moreover, CI and SVI measured in this study were normalized by body surface area for use in future studies. The agreement between CI derived by this method and that by thermodilution [20], Doppler echocardiography [21] and Innocor inert gas rebreathing [22] were reported to be acceptable. We measured cardiac function at high altitude using this device with the idea of future feasibility: i.e., that anyone can measure these parameters any place and at any time. The measurements were carried out for at least 5 min under a steady state following 3 h of rest after the subjects arrived at MFRS. Approximately 1 mL of venous blood was sampled from the cubital vein at 120 m and at high altitude before sleeping. The pH, CO_2_, HCO_3_ and lactate levels were analyzed by iSTAT (Abbot Point-of-Care, Princeton, NJ, USA).

### Statistical analysis

Values are reported as n (%) or median [interquartile range]. Statistical analysis was performed with the Wilcoxon rank-sum test to compare differences between two independent groups and the Wilcoxon matched-pairs signed rank test to compare differences between two paired groups. A *p*-value < 0.05 was considered statistically significant. The data were analyzed using JMP Pro version 14 (SAS Institute Inc., Cary, NC) and are presented using Graph Pad Prism, version 6.04 (GraphPad Software, La Jolla, CA).

## Results

### Change of physiological parameters and venous blood analysis data at high altitude

In this study, 4 of the 11 subjects fulfilled the criteria for AMS. There were no differences in the baseline physiologic parameters and data of venous blood gas analyses between the two groups (Table 1). The change of LLS score is shown in Fig. 1B, and those of SpO_2_, HR and systolic blood pressure are shown in Fig. 1C. The SpO_2_ dropped to around 75% in both groups at MFRS. The CI of the AMS subjects was significantly higher than that of the non-AMS subjects before sleeping (4.9 [4.5, 5.0] vs. 3.8 [3.4, 3.9] mL/min/m^2^; *p* = 0.04) (Fig. 1D). The ΔCI of the AMS subjects was significantly higher than that of the non-AMS subjects measured before sleeping (1.6 [1.4, 2.1] vs. 0.2 [0.0, 0.7] mL/min/m^2^; *p* < 0.01) and after sleeping (0.7 [0.3, 1.7] vs. -0.2 [-0.5, 0.0] mL/min/m^2^; *p* < 0.01) (Fig. 1E). There were no significant differences in pH, CO_2_, HCO_3_^−^ and lactate levels between the AMS and non-AMS subjects at MFRS (Table 2).

**Table 1:** Baseline characteristics

	**AMS (-)**	**AMS ( +)**	***p* Value**
	**(*n* = 7)**	**(*n* = 4)**	
Sex, male, n (%)	3 (42.9)	2 (50.0)	0.819
Age	26 (24–29)	42 (34–42)	0.018*
Body surface area (m^2^)	1.6 (1.5–1.8)	1.6 (1.4–1.7)	0.571
Physiologic parameters			
SpO_2_ (%)	98 (98–99)	98 (97–99)	0.756
HR (bpm)	67 (66–77)	67 (56–76)	0.569
sBP (mmHg)	113 (56–128)	110 (95–121)	0.448
SVI (mL/m^2^)	53 (47–56)	51 (39–52)	0.394
CI (L/min/m^2^)	3.4 (2.9–4.1)	3.0 (2.7–3.5)	0.156
Venous gas analysis			
pH	7.35 (7.35–7.38)	7.37 (7.34–7.40)	0.636
CO_2_ (mmHg)	50.9 (45.9–56.0)	53.7 (52.0–58.9)	0.508
HCO_3_^−^ (mmol/L)	28.7 (27.4–30.9)	31.2 (30.8–31.8)	0.155
Lactate (mmol/L)	0.55 (0.30–0.67)	0.63 (0.47–0.98)	0.298

Data are given as number (%) or median (25th-75th percentile). Significance was determined by Pearson’s chi-squared test or Mann–Whitney U-test

*AMS* Acute mountain sickness, *CI* Cardiac index, *HR* Heart rate, *sBP* Systolic blood pressure, *SpO*_*2*_ Peripheral arterial blood oxygen saturation, *SVI* Stroke volume index

^*^Statistically significant (*p* < 0.05)

**Table 2:** Venous blood gas analysis at the Mt. Fuji Research Station

	**AMS (-)**	**AMS ( +)**	***p* Value**
	**(*n* = 7)**	**(*n* = 4)**	
pH	7.41 (7.37–7.7.44)	7.40 (7.39–7.42)	1.000
CO_2_ (mmHg)	41.5 (40.3–47.8)	44.5 (41.5–50.2)	0.508
HCO_3_^−^ (mmol/L)	27.8 (27.1–28.3)	27.4 (27.0–30.1)	0.155
Lactate (mmol/L)	0.99 (0.63–1.31)	0.87 (0.71–1.95)	0.298

Data are given as number (%) or median (25th-75th percentile). Significance was determined by Mann–Whitney U-test

*AMS* Acute mountain sickness

^*^Statistically significant (*p* < 0.05)

### Changes in LLS, SpO_2_, HR, SVI and CI before and after sleeping at high altitude

**Fig. 2:**
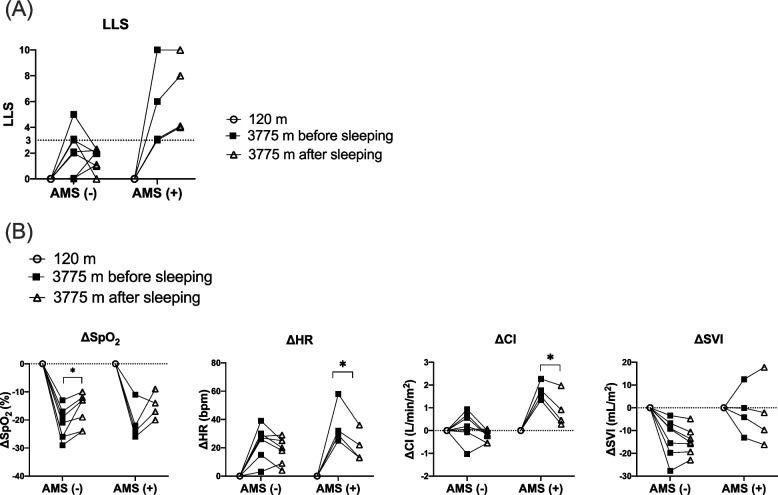
Change in LLS, ΔSpO_2_, ΔHR, ΔCI and ΔSVI before and after sleeping at 3,775 m. **A** Change in LLS. **B** Changes in ΔSpO_2_, ΔHR, ΔCI and ΔSVI. *Indicates a significant difference in each value between before sleeping and after sleeping (*p* < 0.05). All values are expressed as the mean ± SD. *AMS* acute mountain sickness, *CI* cardiac index, *HR* heart rate, *LLS* Lake Louise Score, *SD* standard deviation, *SVI* stroke volume index

At MFRS, LLS improved in 4 of the 7 non-AMS subjects after sleeping, but it did not improve in any of the AMS subjects after sleeping (Fig. 2A). ΔHR and ΔCI dropped in 10 of the 11 subjects following sleeping. Among the AMS subjects, ΔCI after sleeping was significantly lower than that before sleeping (3.8 [3.6, 4.5] vs. 4.9 [4.5, 5.0] mL/min/m^2^; *p* = 0.04) (Fig. 2B).

## Discussion

This study identified a relationship between CI and AMS. Higher values of CI and ΔCI were observed at high altitude in the AMS subjects.

A study measuring CI by electrical cardiometry of subjects arriving at 3,800 m by cable car showed a significant increase in HR, and SVI and CI tended to increase from baseline [19]. A study on Mt. Everest also revealed that HR, SV and CI were increased more at 5,020 m than at sea level [23] as an adaptation to maintain O_2_ delivery [18]. To our knowledge, no study has evaluated CI in subjects exposed to high altitude over a short time as in the present study. Contrary to our hypothesis, high CI was observed in the AMS subjects. Blood lactate before sleeping was within the normal range, suggesting that the subjects developing AMS after sleeping had no metabolic crises before sleeping. However, we did not evaluate lactate levels after sleeping when AMS was diagnosed, so whether lactate levels were elevated in the AMS subjects remains unclear. Further study is needed to clarify the balance between oxygen demand and supply.

Sleeping altitude is reported to be associated with AMS [2], with one reason being the drop in oxygen saturation while sleeping [17]. We evaluated CI before and after sleeping at high altitude and observed that CI had decreased in 8 of the 11 subjects. Another hypothesis might be that high CI was caused by low systemic vascular resistance index (SVRI). Our data showed no differences between MAP with or without AMS. SVRI was calculated as SVRI ∝ (MAP—central venous pressure) / CI. Although not evaluated, if central venous pressure was assumed to be the same between AMS and non-AMS subjects, SVRI was in inverse proportion to CI. A decrease in SVRI was associated with vasodilation, one of the pathogeneses of AMS [24, 25]. Significant decreases in ΔHR and ΔCI were observed in the subjects developing AMS after sleeping. The increased CI was mainly achieved by increasing HR through stimulation of the cardiac β-adrenergic receptors by cardiac sympathetic nerves and circulating adrenaline (epinephrine) [26]. The influence of sleep on the nervous system will also require further evaluation in the future.

A study by Wolsk et al. found that younger participants had higher CI and SVI during exercise compared to older participants, but their hemodynamic parameters were similar at rest [27]. The present AMS subjects who were older than non-AMS subjects showed a higher CI (4.9 vs. 3.8 mL/min/m^2^). An increase in cardiac output at high altitude maintains O_2_ delivery. Further study is needed to determine whether subjects with high cardiac output conditions and age-related cardiac impairment develop AMS.

This study has several limitations. Although this is a pilot study, future studies with a larger sample size are needed. Cardiac function was impaired by age, and AMS subjects were older than non-AMS subjects. Age-related cardiac impairment might have affected the development of AMS.

## Conclusion

Cardiac function was assessed at high altitude. Higher values of CI and ΔCI were observed at high altitude in the AMS subjects. A high cardiac output might be associated with the development of AMS.

## Data Availability

The datasets used and/or analyzed during the current study are available from the corresponding author on reasonable request.

## Abbreviations

AMS: Acute mountain sickness
CI: Cardiac index
HR: Heart rate
LLS: Lake Louise Score
MFRS: Mt. Fuji Research Station
SV: Stroke volume
SVI: Stroke volume index
SVRI: Systemic vascular resistance index
