# Correction: Association between high cardiac output at altitude and acute mountain sickness: preliminary study on Mt. Fuji

**DOI:** 10.1186/s40101-023-00324-5

**Published:** 2023-05-19

**Authors:** Takeshi Ebihara, Kentaro Shimizu, Yumi Mitsuyama, Hiroshi Ogura, Jun Oda

**Affiliations:** Department of Traumatology and Acute Critical Medicine, Osaka University Graduate School of Medicine, 2‑15 Yamadaoka, Suita City, Osaka 565‑0871 Japan

**Correction: J Physiol Anthropol 42, 6 (2023)**

**https://doi.org/10.1186/s40101-023-00322-7**

Following publication of the original article [1], the authors identified an error in Fig. 1E. The legend (AMS(-) n = 7 AMS(-) n = 7) appeared twice in Fig. 1E that causes the overlapping of data.

The original article [1] has been corrected.
